# Comparing the Activity Profiles of Wheelchair Rugby Using a Miniaturised Data Logger and Radio-Frequency Tracking System

**DOI:** 10.1155/2014/348048

**Published:** 2014-04-15

**Authors:** Barry Mason, John Lenton, James Rhodes, Rory Cooper, Victoria Goosey-Tolfrey

**Affiliations:** ^1^Peter Harrison Centre for Disability Sport, School of Sport, Exercise & Health Sciences, Loughborough University, Loughborough LE11 3TU, UK; ^2^Human Engineering Research Laboratories, University of Pittsburgh, Pittsburgh, PA 15206, USA

## Abstract

The current study assessed the validity and reliability of a miniaturised data logger (MDL) against a radio-frequency-based indoor tracking system (ITS) for quantifying key aspects of mobility performance during wheelchair rugby. Eleven international wheelchair rugby players were monitored by both devices during four wheelchair rugby matches. MDL data were averaged over both 1-second (MDL-1) and 5-second (MDL-5) intervals to calculate distance, mean, and peak speeds. The results revealed no significant differences between devices for the distance covered or mean speeds, although random errors of 10% and 12%, respectively, were identified in relation to the mean values. No significant differences in peak speed were revealed between ITS (3.91 ± 0.32 m*·*s^−1^) and MDL-1 (3.85 ± 0.45 m*·*s^−1^). Whereas peak speeds in MDL-5 (2.75 ± 0.29 m*·*s^−1^) were significantly lower than ITS. Errors in peak speed led to large random errors in time and distance spent in speed zones relative to peak speed, especially in MDL-5. The current study revealed that MDL provide a reasonable representation of the distance and mean speed reported during wheelchair rugby. However, inaccuracy in the detection of peak speeds limits its use for monitoring performance and prescribing wheelchair rugby training programmes.

## 1. Introduction

The use of innovative assistive technology within the wheelchair court sports (basketball, rugby, and tennis) has increased dramatically over recent years and has been associated with improvements in athletic performance [1–3]. However, one ongoing challenge faced by researchers and practitioners is the ability to quantify the demands of these sports both accurately and practically in order to optimise how training is prescribed whilst minimising injury risk to athletes.

Miniaturised data loggers (MDL) were originally developed to determine the activity profiles of daily life wheelchair users [4] and have recently been implemented into sporting wheelchair environments [5–7]. The MDL are small, lightweight devices which attach near the axle of the main wheels, powered by long life batteries, enabling data to be collected and stored over periods of approximately 3 months [4]. This has practical implications that may benefit sport science practitioners, as devices could be attached to athletes' sports wheelchair to record the distances covered and speeds reached throughout training sessions over an extended period of time. Practitioners could then review each athlete's performance and modify training programmes accordingly over future training cycles, with minimal input required at individual training sessions.

Sindall et al. [8] recently validated the MDL for use on a court sports wheelchair against a motor-driven treadmill and a 1 Hz global positioning system (GPS). It was revealed that although the MDL provided an accurate and reliable representation of distance at low speeds (<2.5 m·s^−1^), coefficients of variations (% CV) as high as 19.9% CV were reported at speeds in excess of 2.5 m·s^−1^ during controlled laboratory and field based trials [8]. However, when assessing the validity and reliability of a device, it is essential that the device is assessed during movements and intensities specific to the intended activity [9]. In the case of the wheelchair court sports, movements are multidirectional, intermittent activities, which include accelerating, turning, braking, and sprinting [10]. Sindall et al. [8] also attempted to validate the MDL during wheelchair tennis match-play, where significantly lower peak speeds, yet greater total distances and mean speeds, were reported by the MDL in relation to GPS. Unfortunately, the 1 Hz GPS used was unlikely to be sufficient for the assessment of these variables during competition, particularly when high speeds are being reached, questioning its suitability as a valid criterion measure [11–14].

Radio-frequency tracking systems have become increasingly popular for tracking athlete's movements in indoor sports, since they operate in a similar fashion to GPS with the main exception being the use of fixed base stations as sensors instead of satellites. Much like GPS, athletes are required to wear a small lightweight tag, making radio-frequency systems an unobtrusive and practical solution. Previous studies have also demonstrated that these systems can be highly precise and reliable in their assessment of location, distances, and speeds [15–18]. However, unpublished data from our laboratory has validated a radio-frequency-based indoor tracking system (ITS) specifically for use within the wheelchair court sports. It has suggested that at 8 Hz and 16 Hz sampling frequencies, the ITS provides a valid and reliable assessment of distance and mean speed with relative errors ≤ 0.7% observed during a range of tasks specific to the wheelchair court sports. Importantly, even at maximal speeds (>4 m·s^−1^), random errors were <0.10 m·s^−1^, with <2% CV using the ITS, further demonstrating its suitability for use with the wheelchair court sports. Unfortunately, from a practical perspective, the ITS requires greater set-up and calibration time and more practitioner input and are not as portable as MDL, making it slightly less attractive as a monitoring tool in an elite, applied environment.

An accurate quantification of peak speeds is highly desirable given that relative speed zones determined from peak speeds are emerging as a method for monitoring performance and optimising training strategies specific to each individual in team sports [19, 20]. A limitation associated with the MDL is that reed switches are currently positioned at 120° intervals and speed data is calculated as a function of the time and distance between successive reed switch activations, with the latter being determined by the athlete's wheel dimensions [4]. As a consequence, the MDL is not capable of reporting instantaneous speed. Subsequently previous research has averaged speed data over 5-second intervals, to correspond with heart rate data, in order to obtain a “mean” peak speed [6–8]. However, this analysis approach is likely to be too long to establish “true” peak speeds and alternatively averaging MDL data over shorter 1-second intervals may provide a more valid representation of peak speeds.

The aim of the current study was to determine the validity and reliability of the MDL, averaged over both 1-second and 5-second intervals in comparison to a radio-frequency ITS for the quantification of key aspects of wheelchair rugby mobility performance. A secondary aim was to investigate the times and distances spent in specified speed zones, to determine whether the MDL could be an appropriate device for monitoring performance and prescribing training programmes for wheelchair rugby players.

## 2. Methods

### 2.1. Participants

Eleven elite male wheelchair rugby players (age = 26 ± 6 years; body mass = 61.3 ± 10.5 kg) volunteered to participate in the current investigation. All participants were members of a national wheelchair rugby squad. Ethical clearance was approved by the university's local ethical advisory committee and all participants provided their written, informed consent prior to data collection.

### 2.2. Equipment

Participants were tested in their own customised rugby-specific wheelchairs. These wheelchairs varied in mass (16.2 to 19.3 kg), wheel diameter (24 to 25 inch), and camber angle (16° to 19°). Tyre pressures were also self-selected and specific to each individual (110 to 150 psi).

#### 2.2.1. Miniaturised Data Logger

**Figure 1:**
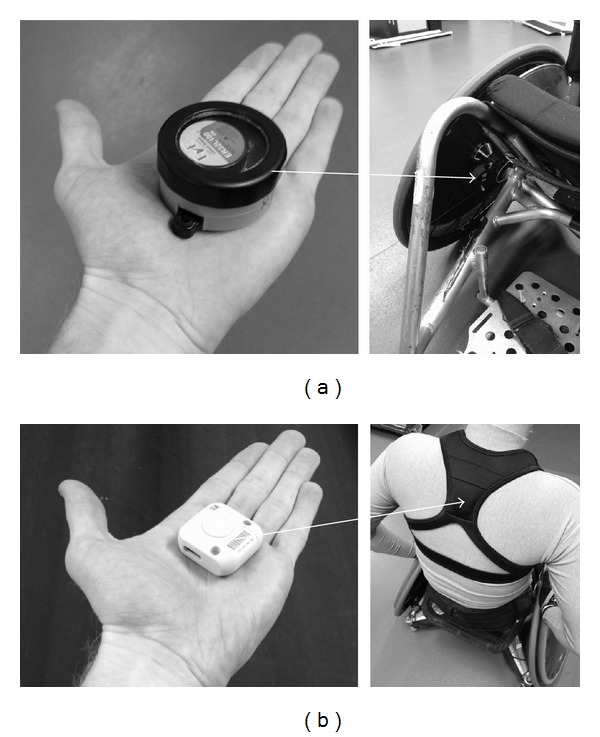
Illustration of (a) MDL and its positioning and attachment on the inside of a wheelchair rugby wheel and (b) a tag and its location in a GPS vest for the ITS.

Two magnetic reed switch MDL were used for each participant during data collection. The MDL, which weighs 96 g, was attached near the axle of both left and right main wheels (Figure 1(a)). As previously described by Tolerico et al. [4], each MDL is powered by a single 1/6D wafer-cell lithium battery. In brief, the MDL measures wheel rotation using three reed switches at 120° intervals, which are attached to a printed circuit board, with a magnet located at the bottom of a pendulum. Each time the wheel rotates, the magnet passes a reed switch and a time stamp is recorded to the nearest 0.10 second. Three reed activations in sequence relate to one wheel rotation. Using the dimensions of each participant's wheel, the following equation was used to calculate distance:

 Distance (m) = No. of reed switch activations · 1/3 wheel circumference [8].

Mean speed was simply calculated as the total distance covered divided by the total playing time. A customised MATLAB programme was used to compute these variables along with peak speed. Within this programme, all variables were analysed over both 1-second (MDL-1) and 5-second (MDL-5) intervals. During MDL-1 peak speed was calculated as the “average” speed for each time stamp activated in 1-second periods. Alternatively the peak speed calculated during MDL-5 referred to the “average” speed of each time stamp across 5-second intervals, as previously adopted by Sindall et al. [6–8].

#### 2.2.2. Indoor Tracking System

The ITS (Ubisense, Cambridge, UK) is a wired, radio-frequency-based tracking system, which provides positioning data in real time. The ITS is comprised of six sensors that communicate wirelessly with small (40 × 40 × 10 mm), lightweight (25 g) tags. The six sensors are positioned high (approximately 4 m) around the perimeter of the court, with each sensor orientated with approximately 40° pitch and exactly 0° yaw. The tags, which are housed in a GPS vest worn by the participants (Figure 1(b)), emit ultrawideband radio-frequency signals. The angle-of-arrival and time-difference-of-arrival of these signals are detected by the sensors to determine an accurate tag location. All tags were set to record at 8 Hz, meaning that a location was obtained for each tag every 0.125 seconds. Raw data were filtered using a 3-pass sliding average filter with a window width proportional to the tag frequency.

### 2.3. Procedures

Data were collected from a total of four simulated wheelchair rugby matches, played over 4 × 8-minute quarters on separate days. Data were collected using the MDL and ITS from a minimum of three to a maximum of five participants at a time. Only data from full quarters were analysed, which gave a total of 42 quarters where data were collected from both devices. During each quarter the total distance covered, mean, and peak speeds were analysed from each device. In addition to this, the time spent and distance covered in the following five relative speed zones were analysed, which were derived using the peak speed (*V*_max⁡_) obtained by both devices [20]:

- <20% *V*_max⁡_—“very low,”
- 20–50% *V*_max⁡_—“low,”
- 51–80% *V*_max⁡_—“moderate,”
- 81–95% *V*_max⁡_—“high,”
- >95% *V*_max⁡_—“very high”.

The absolute time spent and distances covered in 8 arbitrary speed zones ranging from 0 to 4 m·s^−1^ at 0.5 m·s^−1^ intervals was also analysed, as previously defined by Sindall et al. [7].

### 2.4. Statistical Analysis

Data were analysed using the Statistical Package for Social Sciences (SPSS version 21.0). Mean ± SD were calculated for all performance variables collected from both the MDL and ITS. All data were checked for normality using Shapiro Wilk's tests. The mean differences between devices were then explored using either a paired samples *t*-test for normally distributed data or Wilcoxon's signed rank tests where assumptions of normality were violated. Significant differences (*P* < 0.05) identified whether significant systematic bias existed between MDL-1 and MDL-5 in relation to the ITS. To explore the reliability, the absolute differences of each performance parameter between the ITS and both MDL-1 and MDL-5 were first compared to the mean values and checked for normality. A Pearsons correlation was performed on all normally distributed data, whereas Spearmans ranks were performed if conditions for normality were not satisfied to determine if heteroscedasticity was present. Low and insignificant correlations revealed that the current data were homoscedastic meaning that the 95% Limits of Agreement (95% LoA) could be calculated and reported in raw units. Bland-Altman plots were used to investigate the spread of the data with 95% LoA (presented as systematic bias ± random error) used to investigate the reliability of the MDL-1 and MDL-5 for distance covered, mean speed, peak speed, and the times spent and distances covered in arbitrary speed zones. The time and distance in speed zones relative to peak speed were compared between devices using paired sample *t*-tests. Statistical significance was accepted when *P* < 0.05.

## 3. Results

**Figure 2:**
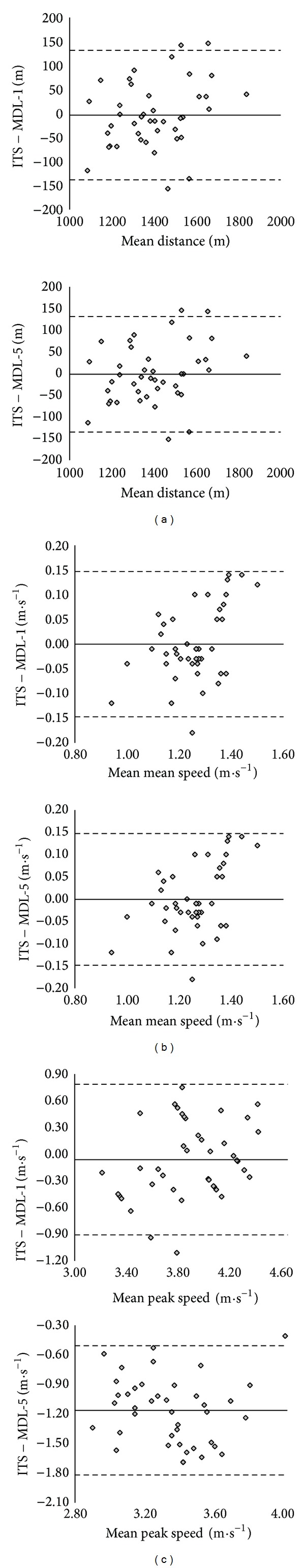
Bland-Altman plots demonstrating the 95% Limits of Agreement between the ITS and MDL-1 (top) and MDL-5 (bottom) for (a) distance covered, (b) mean speed, and (c) peak speed. Solid lines represent systematic bias. Dashed lines represent random error.

No significant differences in distance covered or mean speeds existed between devices (Table 1). Although no significant systematic bias was observed for these parameters between the ITS and both MDL-1 (distance: −3 m, *P* = 0.767; mean speed: 0.00 m·s^−1^, *P* = 0.984) and MDL-5 (distance: −2 m, *P* = 0.875; mean speed: 0.00 m·s^−1^, *P* = 0.951), random error was present. As shown by the Bland-Altman plots in Figure 2, random errors of ±135 m (MDL-1) and ±133 m (MDL-5) were revealed for distance covered, which compared to the mean value (1401 m) represented a relative random error of 10%. Random errors of ±0.15 m·s^−1^ were revealed for both MDL-1 and MDL-5, which equated to 12% random error.

Furthermore in Table 1, peak speeds were significantly lower in MDL-5 compared to ITS and MDL-1 (*P* < 0.0005). The significant systematic bias revealed for peak speed in MDL-5 (−1.16 m·s^−1^) was accompanied by random errors of ±0.66 m·s^−1^ (20%). Although no significant systematic bias was identified for MDL-1 (−0.06 m·s^−1^, *P* = 0.496) random errors of ±0.85 m·s^−1^ existed (Figure 2). This random error reflected 22% of the mean, peak speed value between MDL-1 and the criterion (3.88 m·s^−1^).

**Figure 3:**
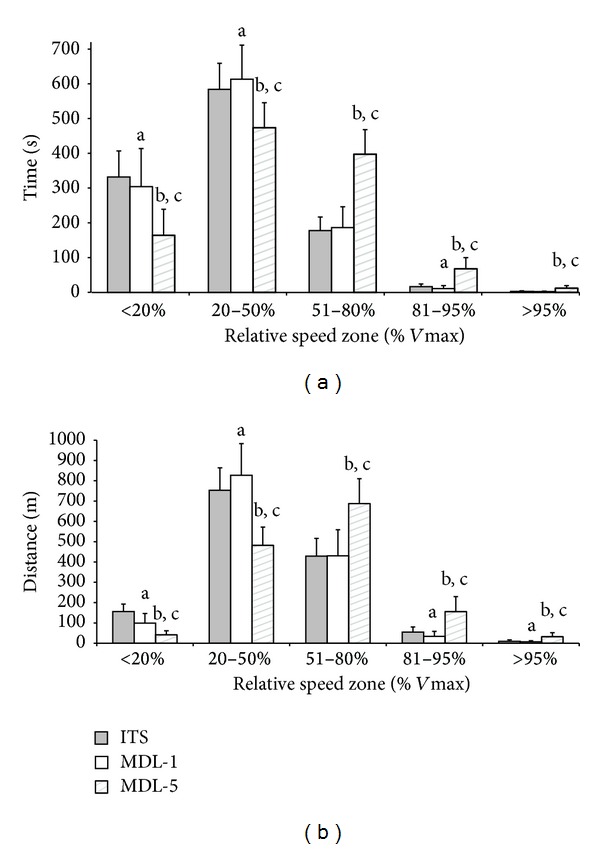
Time spent (a) and distance covered (b) in speed zones relative to the *V*_max⁡_ of each device. All values are mean ± SD. Significant differences between devices are represented by ^a^ITS and MDL-1, ^b^ITS and MDL-5, and ^c^MDL-1 and MDL-5.

Differences in peak speed were shown to influence the time spent and distance covered in the 5 relative speed zones (Figure 3). MDL-5 significantly underestimated the time spent and distance covered in both “very low” and “low” speed zones compared to ITS and overestimated the times and distances in “moderate,” “high,” and “very high” zones (*P* < 0.0005). Significantly less time was spent in “very low” and “high” speed zones and greater time was spent in “low” speed zones for MDL-1 (*P* ≤ 0.035). Distance covered was also significantly reduced in “very low,” “high,” and “very high” speed zones and yet increased in “low” zones for MDL-1 (*P* < 0.0005).

**Figure 4:**
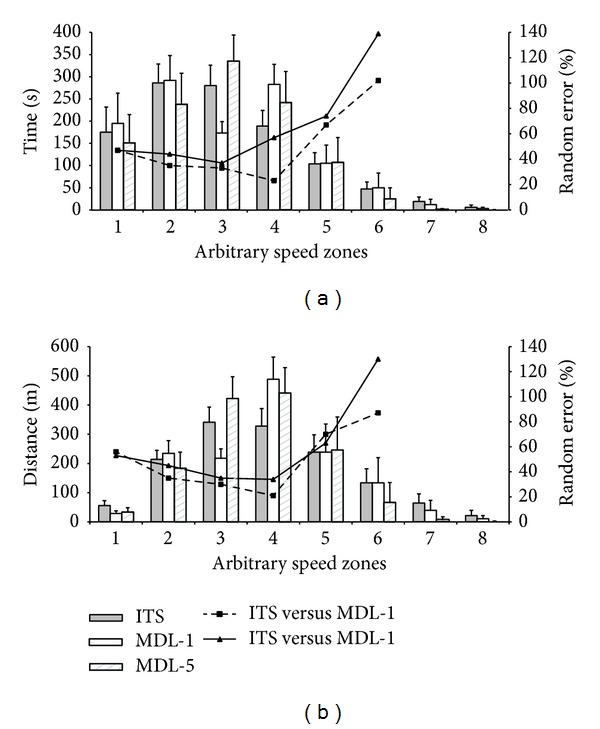
Mean ± SD values for the time spent (a) and distance covered (b) in 8 arbitrary speed zones by each device. The overlaying line graph represents the random error (reported as a % of the mean value). Note that random errors were not calculated for zones 7 and 8 due to insufficient data in these zones for MDL-5. Zones are defined as follows: 1 (0–0.5 m·s^−1^), 2 (0.5–1.0 m·s^−1^), 3 (1.0–1.5 m·s^−1^), 4 (1.5–2.0 m·s^−1^), 5 (2.0–2.5 m·s^−1^), 6 (2.5–3.0 m·s^−1^), 7 (3.0–3.5 m·s^−1^), and 8 (3.5–4.0 m·s^−1^).

The mean time spent and distances covered in arbitrary speed zones independent of *V*_max⁡_ are displayed for each device in Figure 4. MDL-5 demonstrated a significant systematic bias for both the times and distances in each speed zone except for speed zone 5 (Table 2). Less time and distance were reported in speed zones 1, 2, 6, 7, and 8, with greater times and distances revealed in zones 3 and 4 compared to the ITS (Figure 4). Alternatively, MDL-1 only displayed a significant systematic bias for the time spent in zones 1 (*P* = 0.002), 3 (*P* < 0.0005), and 4 (*P* < 0.0005) and the distances covered in zones 1 (*P* < 0.0005), 2 (*P* = 0.001), 3 (*P* < 0.0005), and 4 (*P* < 0.0005). Figure 4 also illustrated the random error experienced between both the MDL-1 and MDL-5 with the ITS for the times and distances in each arbitrary speed zone, with a greater error reported in MDL-5. It was also revealed that the random error for both devices increased as a function of speed, with a sharp increase in error revealed for both times and distances between zones 4 and 5 (Figure 4). However random errors never dropped below 23% for times and 21% for distance in any of the speed zones.

## 4. Discussion

The results of the current study revealed that there was no significant differences between both MDL-1 and MDL-5 and the ITS for the distances covered or the mean speeds reached during competitive wheelchair rugby. No significant difference existed for the detection of peak speeds between MDL-1 and the ITS; however, a systematic bias was revealed for MDL-5, which significantly underestimated peak speeds. Although significant systematic bias was not evident for MDL-1 for the assessment of distance, mean and peak speeds, random errors ranging from 10 to 22% still existed. Similar random errors 10–20% were also observed in MDL-5 for these parameters. These errors had a negative effect on the calculation of time spent and distance covered in both the relative and arbitrary speed zones, particularly for MDL-5, which questions the efficacy of using an MDL for the monitoring and prescription of wheelchair rugby training programmes.

The present findings suggest that the MDL is a suitable device for quantifying the distances covered and the mean speeds of sports wheelchair propulsion. These were the type of parameters that the MDL was initially developed to measure in daily life wheelchair users to gain a basic understanding of the amount of physical activity individuals were performing over relatively long time periods [4]. The current study revealed that during a more dynamic application, such as wheelchair rugby, the MDL still provided a reasonable representation of distances and mean speeds, whereby no significant systematic bias existed. Although random errors up to ±135 m and ±0.15 m·s^−1^ were observed for distance and mean speed, respectively, these errors never exceeded 12% in relation to the mean values. Therefore, it could be argued that sport science practitioners could use the MDL to quantify and monitor the total distance and mean speed during different wheelchair rugby training sessions, on the assumption that the data is interpreted with caution given the magnitude of random error present.

The selection of 1-second (MDL-1) or 5-second (MDL-5) analysis intervals was also not shown to be a key consideration when examining distance covered and mean speed, since no significant differences were observed. This finding is a derivative of how the MDL functions. Since the sum of time stamps counted and the overall time of the activity are all that is used to establish distance and mean speed, the effect that different analysis intervals has is negligible. As Sindall et al. [8] mentioned, any underestimations in distance (and subsequently mean speed) were the likely result of missed time stamps during wheel revolutions. Each time such an event occurs one-third of a wheel circumference is missed from the total distance covered. Although it is unclear as to exactly why this event occurs, this is a limitation currently associated with the MDL. Alternatively there are occasions when the distance measured by the MDL overestimated the distance and mean speed in relation to the criterion, hence the presence of random error. An explanation for this event could not be provided by Sindall et al. [8] during wheelchair tennis applications. However, during wheelchair rugby, where impacts with other wheelchairs form a key part of the sport, it is possible that additional time stamps from the same reed switch are activated as the result of a collision, when in reality the wheel is not revolving. Further investigations would be warranted to determine whether this was the cause of overestimations in data and if so amendments would need to be written into the data processing software. Sindall et al. [8] proposed that the future development of a potential six reed switch MDL may further improve the precision of distance and speed measurements. Such a development has since taken place with a six reed data logger combined with a gyroscope in a “wheel rotation monitor,” although the accuracy of the reed switch device was not investigated [21]. A six reed switch MDL may reduce the frequency of underestimations in distance. For example, if a reed switch fails to register a time stamp, the distance and time before the next possible reed switch will be reduced, as these would now be positioned at 60° intervals as opposed to 120°. However, this development is unlikely to minimise the overestimations observed in distance and mean speed.

Although the use of 1-second or 5-second analysis intervals had no meaningful effect on the distance covered or the mean speed reached, it did have a significant bearing on the detection of peak speed. Systematic bias of −1.16 m·s^−1^ was identified for MDL-5, which demonstrated a significant underestimation of peak speed in relation to the ITS. The rationale for this finding can again be attributed to the way in which MDL data is analysed. For MDL-5, the peak speed at each time stamp within a 5-second period is averaged to ultimately give a “mean” peak speed. This “mean” peak speed is always likely to underestimate the “true,” instantaneous peak speed, since athletes would have to maintain the peak speed for 5 seconds when adopting the approach used by MDL-5. This was clearly unlikely given the intermittent nature of wheelchair rugby, where short duration bursts of high intensity activity are performed repeatedly [5, 10, 22]. This type of error was reduced when data was analysed at 1-second intervals, since no significant systematic bias was observed between the MDL-1 and ITS for the detection of peak speed. By averaging the speed of each time stamp over shorter intervals, mean underestimations of 0.06 m·s^−1^ were observed. Despite this reduction in systematic bias for MDL-1, random errors of ±0.85 m·s^−1^ were still revealed. This finding demonstrated that although MDL-1 slightly underestimated the mean peak speeds, there were individual instances where MDL-1 actually overestimated the peak speed in order to account for the random errors. Again, the rationale for underestimations was clear since the peak speeds were subsequently speeds “averaged” over a 1-second time period. Overestimations in peak speed, which were not anticipated, were likely to be attributed to the way in which speed calculations are influenced by the wheel dimensions and the time stamps between reed switch activations. For example, an athlete with a 24-inch wheel, one reed switch activation, relates to a distance of approximately 0.62 m. If this event occurs over 0.10 seconds, a speed of 6.20 m·s^−1^ will be registered, whereas if it occurs over 0.20 seconds the speed is halved to 3.10 m·s^−1^. Therefore only fixed speeds of relatively large margins, which remain a function of wheel size, are obtainable. A peak speed of 6.20 m·s^−1^ seems highly unlikely based on previous research, which assessed the peak linear speeds of wheelchair rugby players during 15 m sprints [23]. Yet, if in reality the time difference between reed switch activations was in fact 0.14 seconds, the speed registered would have been 4.43 m·s^−1^, which is more feasible. Unfortunately, because the MDL can only register time stamps to the nearest tenth of a second, this activity gets severely rounded up (or down), which is why the designers advise against reporting instantaneous speeds. However, one or two of these “rounded up” speeds within a 1-second interval could easily account for the instances where peak speeds are overestimated in MDL-1. Regardless of the mechanisms responsible for the random error in peak speed, these errors equated to 22% and 20% for MDL-1 and MDL-5, respectively, implying that the use of an MDL, irrespective of analysis intervals, should not be used for the detection of peak speeds. This is due to the fact that large random errors between scientific equipment are a greater concern to researchers than systematic bias since the magnitude and direction of the error is not consistent and subsequently hard to control for [24].

Errors in the detection of peak speed leads to even greater errors when determining the time spent and distance covered in speed zones relative to peak speed. This approach has recently been adopted in able-bodied rugby union, whereby relative speed zones have been implemented to monitor and modify training programmes [19, 20]. The results of the current study suggest that the MDL is not an appropriate tool for similar use in wheelchair rugby. It was revealed that MDL-5 underestimated the time spent and distance covered in “very low” and “low” speed zones, yet it overestimated these parameters in “moderate,” “high,” and “very high” speed zones. Work-rest ratios have previously been defined as the time spent in ≥moderate speed zones (work) in relation to ≤low speed zones (rest), which can be used to plan and monitor the intensity of training sessions [25, 26]. If these principles were applied to the context of the current investigation, MDL-5 would increase the amount of work athletes would be prescribed and reduce their rest. The consequences of this overestimation in workload could potentially place athletes at a far greater risk of injury. Unlike MDL-5, no such pattern was revealed for MDL-1, whereby significant reductions in time and distance were recorded in “very low” and “high” speed zones, whereas increases were revealed in “low” speed zones. Subsequently, work-rest ratios based on this analysis would have appeared much closer to those recommended by the ITS. Despite this, these results must still be interpreted with caution. For instance, the absence of any significant difference between MDL-1 and the ITS at “very high” speed zones was most likely due to the minimal times and distances spent in these zones by athletes (2 ± 2 seconds). Also, given the similarities in total distance between devices, an underestimation of time/distance in one or more speed zones would be counteracted by overestimations in other speed zones. In light of these irregularities, it was clear that MDL averaged over 1 or 5-second would not be appropriate for monitoring athletic performance longitudinally during training sessions.

The errors observed in speed zones relative to peak speed were also inherent within arbitrary speed zones. The results revealed that random errors for the times and distances were relatively high (≥21%) across all speed zones, yet this error clearly increased between zones 4-5 (Figure 4). Zone 5 incorporated speeds ranging from 2.0 to 2.5 m·s^−1^ and therefore this increase in error is not too dissimilar to the 2.5 m·s^−1^ threshold that Sindall et al. [8] reported increases in error to occur with the MDL. Random errors were large for both MDL-1 and MDL-5, although these errors were clearly exacerbated in high speed zones in MDL-5. This was likely to be due to the fact that the “mean” peak speed revealed for MDL-5 was only 2.75 m·s^−1^, so very little activity was registered at zones in excess of this speed. As a result of the limited activity registered by MDL-5 at high speeds, comparisons could not be made between devices at the highest speed zones (zones 7 and 8). Consequently, greater activity was registered in the lower speed zones 3 and 4 (1.0–2.0 m·s^−1^). These findings further reiterate that in its current form, the MDL is suitable solely for the quantification of distance and mean speed. Whenever the time spent and distance covered in speed zones (relative or arbitrary) are of interest an MDL is not an appropriate tool due to its deficiencies at high speeds. As previously mentioned, future developments to the design of the MDL, which include 6 reed switches and a greater frequency for recording time stamps, may increase the precision of the device to make it an effective tool in wheelchair sport settings.

## 5. Conclusions

The current study revealed that the MDL can be considered an acceptable tool for monitoring the distance covered and mean speed during wheelchair rugby applications. However, the MDL is currently not capable of accurately or reliably reporting the peak speeds produced by elite wheelchair rugby players. Consequently, it is not recommended that the MDL is used for the prescription or monitoring of training programmes, since it is also not capable of accurately determining the time spent and distance covered in certain speed zones.

## Figures and Tables

**Table 1:** Differences in performance parameters assessed during full quarters of wheelchair rugby. All values are mean ± SD.

	ITS	MDL-1	MDL-5
Distance (m)	1403 ± 168	1399 ± 187	1401 ± 186
Mean speed (m·s^−1^)	1.26 ± 0.10	1.26 ± 0.13	1.26 ± 0.14
Peak speed (m·s^−1^)	3.91 ± 0.32	3.85 ± 0.45	2.75 ± 0.29^b,c^

Significant differences between devices are represented by

^a^ITS and MDL-1,

^b^ITS and MDL-5,

^c^MDL-1 and MDL-5.

**Table 2:** The 95% Limits of Agreement for the times spent and distances covered in arbitrary speed zones for both MDL-1 and MDL-5 compared to the ITS. The 95% Limits of Agreement are presented as systematic bias ± random error.

Arbitrary speed zones						
	1	2	3	4	5	6
MDL-1						
Times (s)	20 ± 86*	6 ± 101	−107 ± 74*	94 ± 54*	1 ± 70	3 ± 49
Distances (m)	−27 ± 24*	21 ± 78*	−124 ± 84*	160 ± 86*	0 ± 167	0 ± 117
MDL-5						
Times (s)	−24 ± 77*	−48 ± 116*	55 ± 114*	53 ± 122*	3 ± 78	−22 ± 50*
Distances (m)	−22 ± 24*	−29 ± 89*	81 ± 135*	113 ± 132*	7 ± 152	−68 ± 130*

*denotes a significant systematic bias in relation to ITS. No statistical tests were performed for zones 7 and 8 due to the insufficient sample of athletes who registered speeds in these zones for each device.
